# Supplementary material for: Nanogold-Carried Graphene Oxide: Anti-Inflammation and Increased Differentiation Capacity of Mesenchymal Stem Cells
Source: Nanomaterials (Basel). 2021 Aug 11;11(8):2046. doi: 10.3390/nano11082046 (PMC8398640; doi:10.3390/nano11082046)
Supplement: Supplementary file 1 [file nanomaterials-11-02046-s001.zip › nanomaterials-1323228-supplementary.pdf]

Figure S1

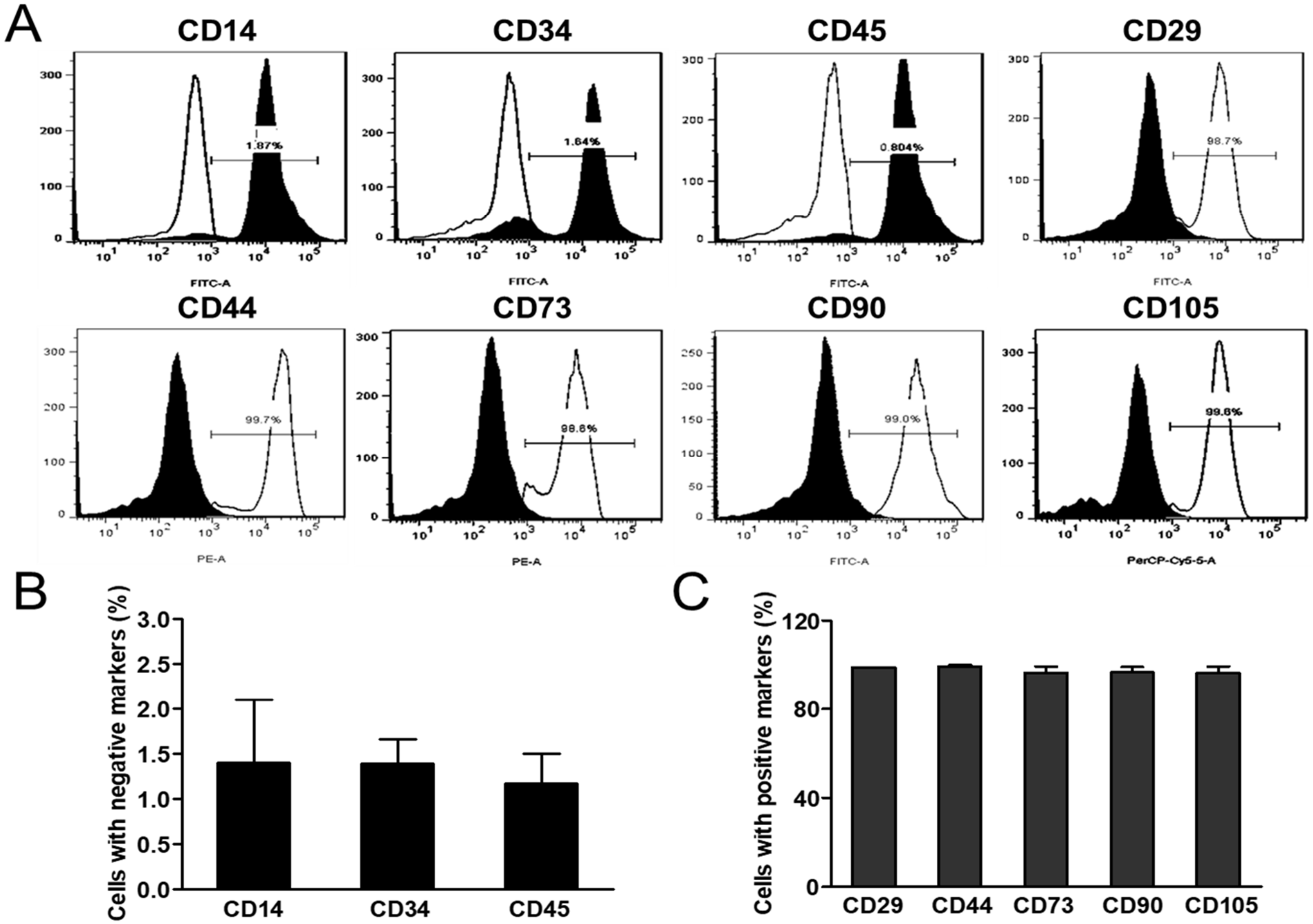

Figure S1. Characterization of MSCs using a flow cytometry analysis. (A) Cells were harvested and incubated with the respective antibody conjugated with fluorescein isothiocyanate (FITC), phycoerythrin (PE) and/or PerCP-Cy5.5, against the indicated markers: CD14-FITC, CD29- FITC, CD34-FITC, CD44-PE, CD45-FITC, CD73-PE, CD90-FITC, and CD105-PerCP-Cy5.5 (BD Pharmingen, USA). Filled area represents isotype controls. (B) The quantification of CD14, CD34 and CD45 by Flow Cytometry. (C) The quantification data of CD29, CD44, CD73, CD90 and CD105 expression.

Figure S2

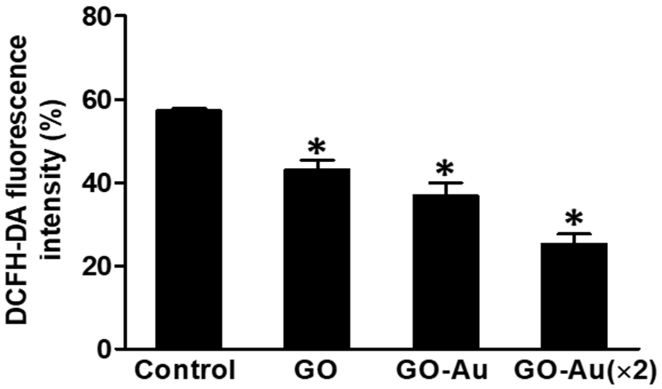

Figure S2. Effects of different materials on intracellular ROS generation. (A) The MSCs were cultured on various nanomaterials of GO, GO-Au and GO-Au (×2) for 24 h. The cells were incubated with 10 μM of DCFH-DA for 30 min, and then harvested and subjected to ROS generation using a flow cytometry analysis. (B) The intracellular ROS generation was recorded and quantified through detected the DCFH-DA fluorescence intensity. The data were expressed as means ± SD of three independent experiments. * $p < 0.05$.

Figure S3

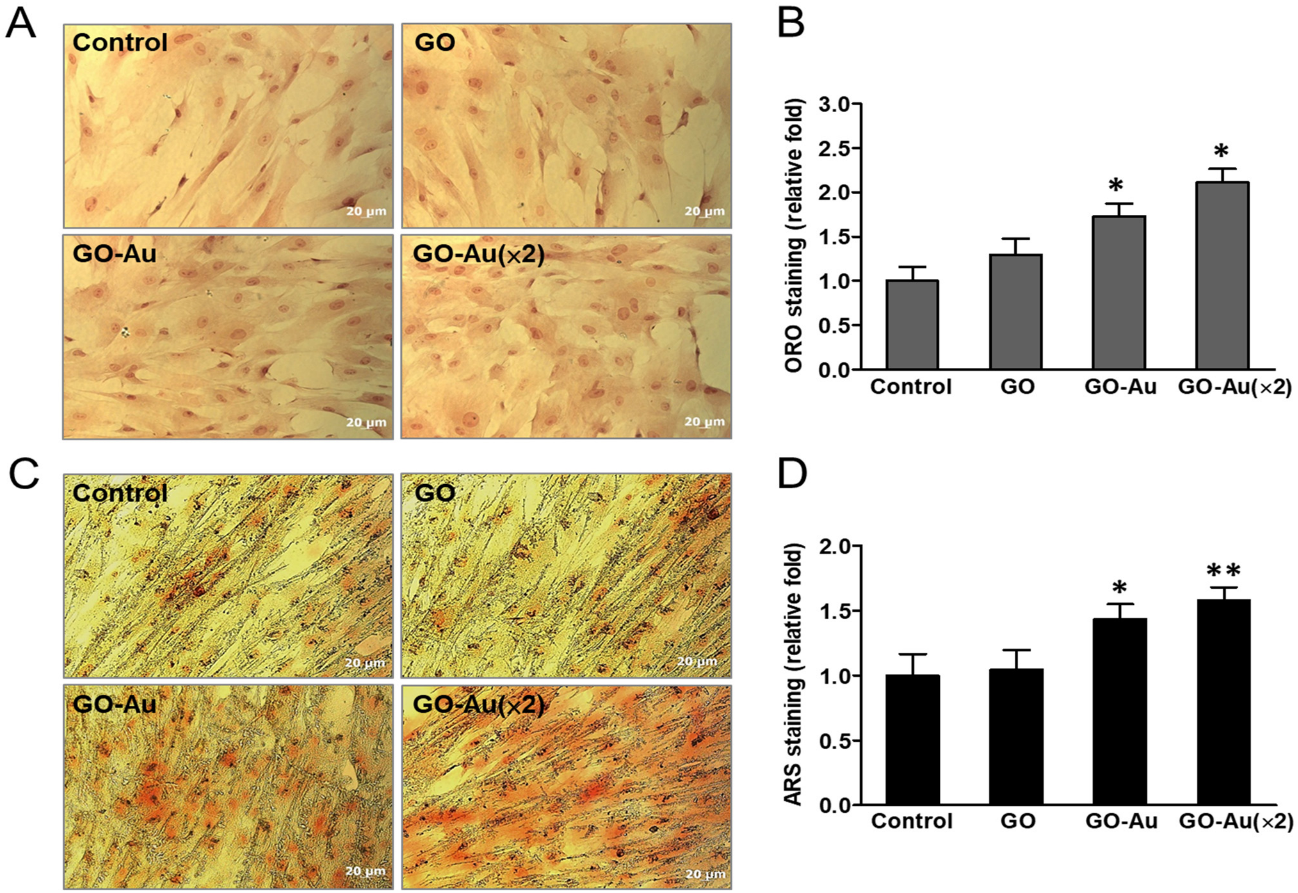

Figure S3. Characterization of GO-Au-mediated MSCs differentiation into adipocytes and osteocytes. Cells were cultured on various nanomaterials of GO, GO-Au and GO-Au (×2) for 7 days. MSCs were then estimated (A-B) the adipocyte differentiation and (C-D) the osteogenic differentiation using Oil-Red O (ORO) staining assay and Alizarin Red S (ARS) staining analysis, respectively. The number of ORO-positive adipocytes was counted and recorded from a given area on the culture slides. The quantification of osteogenic differentiation was obtained from absorbance of the de-stained ARS supernatant and measured at 450 nm using the microplate reader. Data were presented as the mean ± SD, * $p < 0.05$, ** $p < 0.01$.
